# The Diabetes teleMonitoring of patients in insulin Therapy (DiaMonT) trial: study protocol for a randomized controlled trial

**DOI:** 10.1186/s13063-022-06921-6

**Published:** 2022-12-07

**Authors:** Stine Hangaard, Thomas Kronborg, Ole Hejlesen, Tinna Björk Aradóttir, Anne Kaas, Henrik Bengtsson, Peter Vestergaard, Morten Hasselstrøm Jensen

**Affiliations:** 1 Steno Diabetes Center North Denmark, Mølleparkvej 4, 9000 Aalborg, Denmark; 2 Department of Health Science and Technology, Aalborg University, Fredrik Bajers Vej 7C, 9220 Aalborg Ø, Denmark; 3 Novo Nordisk A/S, Novo Alle 1, 2880 Bagsværd, Denmark; 4 Department of Endocrinology, Aalborg University Hospital, Aalborg, Denmark; 5 Department of Clinical Medicine, Aalborg University, Aalborg, Denmark

**Keywords:** Diabetes, Insulin, Telemedicine, Telehealth, Telemonitoring, CGM, Adherence

## Abstract

**Background:**

The effect of telemedicine solutions in diabetes remains inconclusive. However, telemedicine studies have shown a positive trend in regards to glycemic control. The telemedicine interventions that facilitate adjustment of medication seems to improve glycemic control more effectively. Hence, it is recommended that future telemedicine studies for patients with diabetes include patient-specific suggestions for changes in medicine. Hence, the aim of the trial is to explore the effect of telemonitoring in patients with type 2 diabetes (T2D) on insulin therapy.

**Methods:**

The trial is an open-label randomized controlled trial with a trial period of 3 months conducted in two sites in Denmark. Patients with T2D on insulin therapy will be randomized (1:1) to a telemonitoring group (intervention) or a usual care group (control). The telemonitoring group will use a continuous glucose monitor (CGM), an insulin pen, an activity tracker, and smartphone applications throughout the trial. Hospital staff will monitor the telemonitoring group and contact the subjects by telephone repeatedly throughout the trial period. The usual care group will use a blinded CGM the first and last 20 days of the trial and will use a blinded insulin pen for the entire period.

The primary endpoint will be changed from baseline in CGM time in range (3.9–10.0 mmol/L) 3 months after randomization. Secondary endpoints include change from baseline in glycated hemoglobin (HbA1c), total daily dose, time above range, and time below range 3 months after randomization. Exploratory endpoints include health-related quality of life, diabetes-related quality of life, etc.

**Discussion:**

The DiaMonT trial will test a telemonitoring setup including various devices. Such a setup may be criticized, because it is impossible to determine which element(s) add to the potential effect. However, it is not possible and counterproductive to test the elements individually, since it is the full telemedicine setup that is being evaluated. The DiaMonT trial is the first Danish trial to explore the effect of telemonitoring on patients on insulin therapy. Thus, the DiaMonT trial has the potential to form the basis for the implementation of telemedicine for patients with T2D in Denmark.

**Trial registration:**

ClinicalTrials.gov NCT04981808. Registered on 8 June 2021.

## Introduction

### Background and rationale

Diabetes is a major problem for the global health. In 2017, it was estimated that 8.4% of the adult global population had diabetes. This number is expected to increase to approximately 9.9% (425 million) in 2045 due to an increase in unhealthy diets, obesity, physical inactivity, etc [1–4]. Approximately 90-95% of patients with diabetes have type 2 diabetes (T2D) [5, 6].

Maintaining optimal glycemic control is crucial for both the prevention and control of diabetes-related complications [7]. However, optimal glycemic control is difficult to maintain. This is primarily due to the fact that it is challenging to estimate the correct dose of diabetes medication in order to avoid hypo- and hyperglycemia [7, 8]. Large US studies indicate that less than 50% of patients with diabetes reach their treatment goals [9, 10]. This suggests that the results obtained in research are not reflected when observing patients in the real world [11]. An important reason for this is that patients do not adhere to the prescribed medical treatment [11]. Therefore, medical treatment may be supplemented by telemedicine with an eye to achieving the desired treatment goals.

In telemedicine, technology is used to support patients with diabetes in disease management over a distance [12, 13]. The telemedicine solutions are very diverse and can range from simple reminders via Short Message Service (SMS) to more complex solutions where the patient does various measurements at home, which are monitored over the distance by a health care professional [14–20]. As diabetes care is primarily handled outside a hospital setting in Denmark and other countries, the potential of telemedicine in supporting patients with diabetes in achieving their treatment goals is promising. In addition, telemedicine may be relevant for those patients who are limited in their opportunities for physical attendance for a variety of reasons [21].

In a 2017 systematic review, it was concluded that telemedicine solutions are a safe way to provide support for self-care for patients with diabetes [22]. Moreover, the use of telemonitoring is cost-effective in diabetes management [23]. In contrast, previous reviews on the effect of telemedicine solutions have shown divergent results, though with a positive trend in regards to glycemic control [13, 24–27]. A recent comprehensive review and meta-analysis by Faruque et al. [13] showed an improvement in glycated hemoglobin (HbA1c) in patients who offered telemedicine as a supplement to regular therapy. The results indicated that future telemedicine studies for patients with diabetes should include tailored patient-specific suggestions for changes in medicine, as the telemedicine interventions facilitating adjustment of medication improved glycemic control more effectively [13]. Thus, there is a need to develop a telemedicine solution with the possibility to customize the treatment depending on the individual patient and medical needs.

The Adherence through Cloud-based Personalized Treatment for Type 2 Diabetes project (ADAPT-T2D) has an overall objective of improving treatment goals for patients with type 2 diabetes. This has endeavored through the development and testing of a telemedicine solution for patients with type 2 diabetes who are on insulin therapy. The first trial in the ADAPT-T2D project is the *Dia*betes tele*mon*itoring of patients in insulin *t*herapy (DiaMonT) trial. It is particularly difficult for patients on insulin therapy to adhere to treatment goals, as a number of barriers are associated with insulin therapy [28]. Such barriers may be related to the patient, medication factors, or system factors. For instance, patients may forget or fear to take medication, the medication regimen may be very complex, expensive, or have side effects, and the patient support may be inadequate [28, 29]. Therefore, this particular group of patients is the population of interest in the present trial.

### Objectives

The aim of the trial is to explore the effect of telemonitoring in patients with T2D on insulin therapy.

### Trial design

The design is an open-label randomized controlled trial with a trial period of 3 months. Patients with T2D in telemonitoring (intervention) are compared with patients with T2D in usual care (control). Neither the included patients nor the clinical staff involved in the trial can be blinded to assignment as they will know whether a patient is being telemonitored or not. The data analysis will be performed by researchers from the project group, who will not be blinded to group assignments for practical and financial reasons.

The trial will be carried out in accordance with the Helsinki Declaration and the principles of good clinical practice (GCP). The trial has been approved by the Regional Ethical Committee of North Jutland (N-20200068).

## Methods: Participants, interventions, and outcomes

### Study setting

The trial will be conducted in two sites in Denmark: Steno Diabetes Center North Denmark (Aalborg University Hospital) and Steno Diabetes Center Zealand (Nykøbing Falster Hospital).

### Eligibility criteria

We aim to include 400 participants. The participants will all be patients with T2D who are already treated with insulin. The participants will receive an insulin pen from Novo Nordisk A/S, which only works with Novo Nordisk products. Therefore, participants who are not treated with Novo Nordisk insulin will be shifted to Novo Nordisk basal insulin (Tresiba®) by trial initiation. If the participants should have any problems, concerns, or questions related to the shift in insulin, a telephone hotline with trained staff will be available for the participants 24/7.

Participants will be included based on the following criteria: Women and men ≥ 18 years, T2D diagnosis for ≥ 12 months, residence in Region North Denmark or Region Zealand, in treatment with insulin, being able to use a smartphone along with the other devices to be used in the trial, and able to understand and read Danish. Diagnosis of T2D was defined as either (1) glycated hemoglobin A1c ≥ 48 mmol/mol; (2) venous plasma glucose ≥11.1 mmol/l incl symptoms of polyurea, polydipsia, unexplained weight loss, or recurrent infections; or (3) venous plasma glucose ≥ 7.0 mmol/l or a 2-h plasma glucose ≥ 11.1 mmol/l after an oral glucose tolerance test. Participants will be excluded based on the following criteria: pregnancy or breastfeeding, major surgery planned during the trial period, participation in other trials, and terms that, in the opinion of the investigator or subinvestigators, render the participant unfit to conduct the trial, including lack of understanding of the trial or lack of physical or cognitive ability to participate.

### Recruitment

The participants will be recruited by use of multiple approaches. First of all, the participants will be recruited at Aalborg University Hospital and at Nykøbing Falster Hospital when attending endocrinology clinic visits. For recruitment purposes, posters will be displayed at the endocrinology clinic at both hospitals with information about the trial. Hospital staff will hand out a recruitment leaflet to interested patients. When visiting the endocrinology clinic, patients can consent to being contacted by representatives from the ADAPT-T2D project team in order to receive more detailed information about the trial. Additionally, a patient group has consented that they may be contacted in connection with initiation of new trials at the endocrinology clinic at Aalborg University Hospital. This group of patients will be contacted by phone and will later receive further information about the trial by mail if interested. Moreover, the recruitment will take place through advertising in newspapers, in the Danish diabetes magazine, on social media, in relevant patient organizations, and on the websites of the involved Danish regions.

### Informed consent

Interested patients will be called in for an information interview with the possibility of a companion. This is described in the participant information letter, which is sent or handed over to the patient prior to the interview. The participant information letter also explains the purpose and design of the trial. The information interview takes place at Aalborg University Hospital in a private room. The information interview is conducted by the investigator, a subinvestigator, or a delegated project team member with the necessary professional knowledge. During the interview, the participant will be made aware of the possibility of reflection time prior to giving informed consent. Consent can be withdrawn at any time and without justification. Only when the informed consent has been obtained with the signature of both the participant and the investigator or a subinvestigator, the trial can begin.

### The randomization process

At the beginning of the trial, the participant will be randomized to either the telemonitoring group or the usual care group during a visit to the endocrinology clinics at Aalborg University Hospital or Nykøbing Falster Hospital. Randomization will be performed in RedCap (2020 Vanderbilt University) based on a computer-generated random list (ratio 1:1). The list will be concealed in RedCap until interventions are assigned. Prior to randomization, the participants will be informed about the trial and sign informed consent. Medical laboratory technicians, nurses, and researchers will enroll the participants, give them information regarding the trial, obtain informed consent, and assign the participants to interventions. The trial is open-labeled, since both participants and staff involved in the trial will know whether the participants are being telemonitored or not.

Basic baseline information about the participants will be obtained at trial start in addition to questionnaire data (Table 1). Moreover, a venous blood sample will be drawn to cover secondary endpoints as well as baseline information. A lab technician/nurse will be responsible for the blood sampling.

**Table 1:**
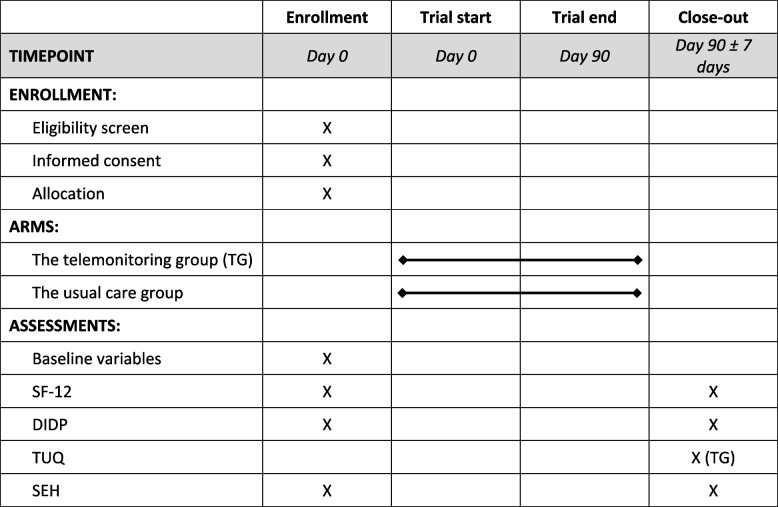
The deployment of questionnaires in the two study groups

*Abbreviations: SF-12* short form 12 (measuring health-related quality of life) [30], *DIDP* DAWN2 Impact of Diabetes Profile (measuring the perceived impact of diabetes) [31], *TUQ* telemedicine usability questionnaire (measuring the quality of the telemedicine intervention) [32], *SHE* Subjective Experience Health questionnaire [33]

### The intervention

#### The telemonitoring group

The telemonitoring group will be provided with a continuous glucose monitor (CGM), a new smart pen from Novo Nordisk A/S, an activity tracker, and a smartphone (unless the participant prefers to use his/her own smartphone). The smart pen will be used for long-acting insulin for all participants. A second smart pen will be provided to those participants who are also on short-acting insulin. The participants will be trained to use the technologies provided at trial inclusion.

The telemonitoring group will use the distributed devices continuously to collect, log, and transfer tissue glucose levels, insulin administration, activity, and sleep at home for the entire trial duration. Laboratory technicians/nurses affiliated with the endocrinology clinics will perform the monitoring. The frequency of monitoring is tailored to the needs of each individual participant. The monitoring laboratory technicians/nurses will contact the participant by phone at least three times — at 1 week, 1 month, and 2 months after inclusion in the trial. Participants may be contacted on an ongoing basis if it is considered relevant by the monitoring lab technicians/nurses. The first phone call, after 1 week, is performed with an eye to ensuring that the participants have started the monitoring successfully and understand how to use the devices. During the calls after one month and 2 months after inclusion, the monitoring lab technicians/nurses will talk to the participants about their data from the past month. The ongoing calls are expected to have a more specific focus. Such calls will be performed if the lab technicians/nurses monitor data that they consider to be unusual. The frequency of such calls will thus rely on the individual assessment of the lab technicians/nurses, as they are not supported by any decision support algorithms. The monitoring lab technicians/nurses may give treatment advice during all calls and potentially change insulin doses based on the monitored data (after consulting a doctor). The participants are welcomed to call the lab technicians/nurses all weekdays during the intervention period if they have any questions regarding their data. All calls to participants are recorded in the participants’ respective journals.

For technical challenges, participants can contact research staff at Steno Diabetes Center by phone via a technical support line.

#### The usual care group

The usual care group will receive standard of care in accordance with Danish guidelines. In addition, a new Novo Nordisk insulin smart pen is provided to all participants in the usual care group. The smart pen will be used for long-acting insulin for all participants. A second smart pen will be provided to those participants who are also on short-acting insulin. The pens received by the usual care group will be blinded to ensure that participants are unable to see their data. The pens will collect data used for later analysis and comparison of adherence to insulin prescription in the intervention group and the control group. In addition, the usual care group will be provided with a continuous glucose monitor (CGM), which they will be asked to wear the first and last 20±2 days of the trial period. The CGM will also be blinded so that the participants are unable to see their data. The CGM data will be used for analysis of the primary and secondary outcomes. Thus, participants in the usual care group will receive usual care with an addition of the blinded devices. No changes in treatment, including medicine/dosage, will be performed based on the blinded devices, as neither patients nor clinical staff will have access to the data. The control group will not be compensated financially for their participation in the trial. However, by the end of trial, the monitoring lab technicians/nurses will offer to print out the data from the blinded devices and go through them with the participants in the usual care group.

The authors hypothesize that the telemonitoring intervention will significantly improve time-in-range compared to usual care.

#### Criteria for discontinuing

The trial will be terminated in the event of any serious adverse events related to the trial as considered by the primary investigator. The trial is stopped for each participant if severe hypoglycemia, ketoacidosis, or severe hyperglycemia is recorded and related to the trial based on the primary investigator’s assessment.

#### Strategies to improve adherence to intervention

Adherence to the intervention will be monitored based on the data received from the devices.

#### End of trial

**Fig. 1:**
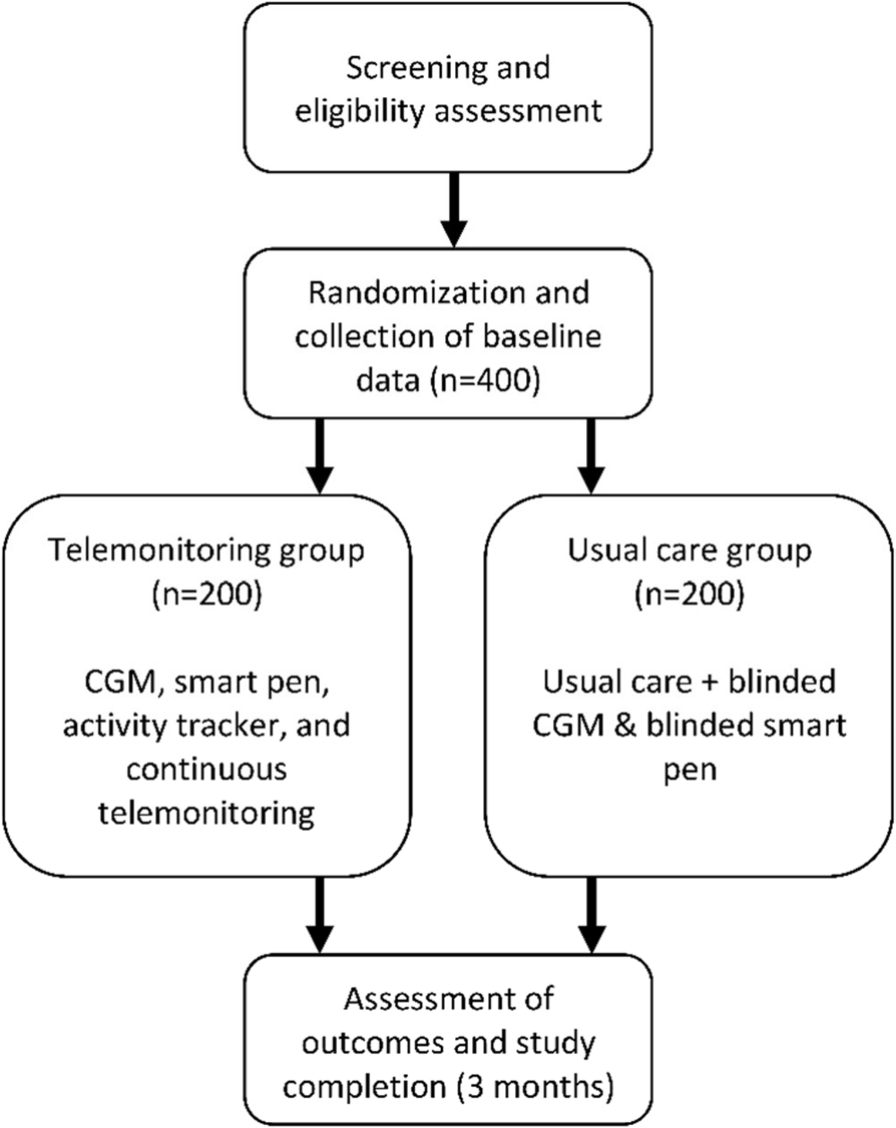
The 3-month flow of the trial

At end of trial, participants from both groups visit the endocrinology clinic again. The participants are encouraged to answer various questionnaires and a laboratory technician/nurse will take a venous blood sample. The flow of the trial design is illustrated in Fig. 1.

### Outcome measures

#### Primary endpoint

Change from baseline in CGM time in range (3.9–10.0 mmol/L) 3 months after randomization.

#### Secondary endpoints

1. Change from baseline in HbA1c 3 months after randomization
2. Change from baseline in total daily dose 3 months after randomization
3. Change from baseline in CGM time in time below range (≤3.9 mmol/L) 3 months after randomization
4. Change from baseline in CGM time in time above range (>10.0 mmol/L) 3 months after randomization

#### Exploratory endpoints

The exploratory endpoints are based on questionnaires and monitoring data, and include the following:

5) Change from baseline in CGM endpoints 3 months after randomization: number of days worn, percentage of time active, mean glucose, glycemic variability, time in hyperglycemia (>13.9 mmol/L), time in hypoglycemia (<3.0 mmol/L), episodes (hypoglycemia and hyperglycemia) 15 min and area under the curve.
6) Use of the telemonitoring equipment: The frequency of use of the telemonitoring equipment during the trial is assessed directly from the delivered devices.
7) Telemonitoring usability: Data is collected via the Telemedicine Usability Questionnaire. The telemedicine group will answer the questionnaire at the 3-month assessment.
8) Diabetes-related quality of life: Between group differences measured by the DIDP questionnaire at baseline and at the 3-month assessment.
9) Quality of Life: Between group differences measured by the SF-12 questionnaire at baseline and at the 3-month assessment.
10) Adherence to insulin dosing and timing. Any differences in the use of insulin between the two groups are examined based on data from the insulin pens.
11) Number of hypoglycemic events 3 months after randomization.

In addition, qualitative interviews will be conducted with selected participants in order to gain deeper insight into the participants’ experience of telemonitoring.

Hospital staff will make appointments with the participants for the final visit, during which the final data will be collected. Should a participant not show up for the appointment, hospital staff will contact the participant by telephone to schedule a new appointment. Participation in the final visit is considered to be completion of trial regardless of the degree of use of technologies. The trial ends after the last participant’s final visit.

#### Data management

Data will be archived both manually and on a computer in RedCap. Signed statements of informed consent as well as completed questionnaires are stored and kept locked. Questionnaire replies and other relevant patient data are entered and stored in RedCap. There will be access protection on both computer and used storage media, which is known only by the investigator, subinvestigator, and relevant members of the project team. Review and manipulation of third party data will only take place by agreement between the subinvestigator and the primary investigator. Data will be stored for the period notified to the Danish Data Protection Agency. In the event of a request for renewal, the trial will be reassessed for the purpose of permission. Data cleaning is performed during the entire conduction of the trial. Checks include cross-validation of dates, search for duplicates, and source data verification and are fired on collected data. Correction of source data follows ICH guidelines for good clinical practice.

#### Confidentiality

Information about the participants will be obtained during trial inclusion. This information includes health status, medication, and comorbidities. This information is collected for two reasons. First, the information is obtained to ensure the suitability of the subjects in relation to inclusion in the study. Second, the information along with demographic data must be used for later analysis in an anonymized form. No information will be obtained until the participant has given her/his consent. This consent gives the primary investigator, subinvestigators, and delegated project team members direct access to obtain information in the participants’ journal, including the electronic journal. The obtained information will include the participants’ health conditions, which is necessary in order to complete the trial and for the purpose of control, including self-regulation, quality control, and monitoring. Only staff affiliated with Steno Diabetes Center will have access to the information in the patients’ journal. Other parties in the ADAPT consortium will not have access to any personally identifiable data.

#### Plans for collection, laboratory evaluation, and storage of biological specimens

As described, a blood sample is drawn at the beginning and at the end of the trial for all included subjects. This is initially done for analysis of the following: HbA1c, lipids, c-peptide, and insulin. Approx. 60 ml will be drawn per sample. All samples will be analyzed immediately after extraction at Aalborg University Hospital, after which the samples will be destroyed.

A research biobank is set up to collect material for later analysis of parameters, which were not possible to predict before the start of the trial. This specifically includes cardiovascular risk and bone disease associated with glucose variation. These factors include:

- P1NP
- CTX
- Sclerostin
- Glucagon
- Plasma-ionized calcium
- Plasma albumin-adjusted calcium

All blood samples will be taken and handled by lab technicians/nurses at SDCN or SDCS during the initial and final visit. All biological material is destroyed at the end of the project when all analyses related to the specific research project have been completed.

### Statistical analysis

Sample size calculation is based on the following formula:

$$n=\frac{2{\sigma_d}^2}{\tau^2}{\left({z}_{a/2}+{z}_{\beta}\right)}^2$$

*τ* is the minimum detectable difference in mean and *σ*_*d*_ is the standard deviation of the difference. Based on an assumed difference in reduction of CGM time in the range of 80 min (*τ* =  − 80), a standard deviation of the difference of 220 min (*σ*_*d*_ = 220) [34], a significance level of 0.05 and a power of 0.9, the number of participants is 320. With an estimated dropout rate of 25%, 400 participants must be included. The large dropout rate is primarily expected in the usual care group. To minimize loss to follow-up, the trial staff will provide the participant in the usual care group with a printout of their data at study completion and offer a consultation regarding the data.

A variety of statistical methods will be applied to investigate the objectives of the trial after end of trial. No analysis is planned during the data collection period. All statistical methods will be applied on the Full Analysis Set that includes all randomized participants. Imputation of missing data is described below.

Change in CGM metrics and total daily insulin dose will be calculated as the difference between mean of the metric, e.g. time in range, or total daily insulin dose the 2 weeks before end of trial and the 2 weeks after randomization. Using data from all patients, the primary endpoint will be investigated with the following primary analysis. The primary analysis is performed with a statistical model that includes multiple imputation, where patients without CGM at scheduled visits get their CGM time in range imputed. An analysis of variance model with the region as a factor and CGM time in range at baseline as a covariate is used to estimate the effect of telemonitoring on CGM time in range. Sensitivity analysis with similar configurations will be applied to subgroups.

The secondary endpoints will be investigated using statistics, models, and algorithms that include differential equations in compartment modeling, as well as algorithms in Machine Learning.

All participants included in the trial will be included in the statistical analyzes. The statistical significance level of 5% is used in the trial (*p* < 0.05). Participation in the final visit is considered to be completion of trial regardless of the degree of use of technologies. The trial ends after the last participant's final visit.

### Monitoring

#### Composition of the coordinating centre and trial steering committee

The steering committee of the ADAPT-T2D consortium will oversee the trial and serve as the data monitoring committee. Furthermore, a research lab technician from Aalborg University Hospital, who are not directly involved in the trial, will perform audit on the data quality throughout the trial period.

The core group running the trial will consist of lab technicians and a research project manager. Besides continuous meetings throughout the trial period (when necessary), the core group will have monthly status meetings. The primary investigator, the data auditing lab technician, and other relevant project members will also be invited to these meetings and attend when relevant.

#### Adverse event reporting

There are no expected harms related to the trial. Any unexpected adverse events related to the trial are recorded in the electronic patient journal and in the trial master file in RedCap with information on whether the primary investigator attributes association to the trial. All adverse advents associated to the trial will be reported in the primary trial publication.

## Discussion

The DiaMonT trial is designed to explore the effect of telemonitoring in patients with T2D on insulin therapy. Several studies have already explored the efficacy of various telemedicine interventions in diabetes [13, 24–26]. However, the majority of the existing telemedicine studies in T2D do not focus on patients in insulin therapy [13, 35–45]. Telemedicine interventions that include medication adjustment have been shown to improve glycemic control more effectively than interventions without medication adjustment. Thus, telemedicine studies that include tailored patient-specific suggestions for changes in medicine have been called for [13], underlying the need for trials such as the DiaMonT trial.

The DiaMonT trial will test a telemonitoring setup, which includes various devices. Such a setup has limitations, because it is not possible to determine which element(s) of the telemedicine setup add to the potential effect. However, it is not possible to test each device separately, and it may not make sense, since it is the full telemedicine setup that is being evaluated. Moreover, there may be interactions in-between the different devices and it would require a very exhaustive trial design to test such potential interactions. In general, telemedicine solutions are complex and context-dependent [46], which also apply to the DiaMonT trial. The trial is further limited by the fact that it is open-label. However, it would be impossible to blind the participants as well as the monitoring lab technicians/nurses. Moreover, it is a limitation that the participants in the usual care group will wear a CGM. Even though the CGM is blinded, one may assume that the participants in the usual care group may change their behavior due to the CGM, because they know that their glucose data are being collected.

A potential practical issue in the trial is the blinding of devices for the usual care group. The devices will be blinded manually using a black pen or a piece of black tape. If a participant in the usual care group is determined to see his/her data on the blinded devices in spite of being told not to, it will be difficult to keep the device blinded.

To the best of our knowledge, the DiaMonT trial is the first Danish trial to explore the effect of telemonitoring in patients with T2D on insulin therapy. The trial has the potential to form the basis for the national implementation of telemedicine for patients with T2D in Denmark. National implementation of telemedicine in Denmark has begun for COPD [47], and the experiences and results from the DiaMont trial would contribute to a prospective national implementation of telemedicine for patients with T2D in Denmark.

### Trial status

The present protocol is version number 1, 30.6.2021. Recruitment is expected to begin July 1, 2021. Recruitment is expected to be completed by July 1, 2022.

## Data Availability

Only staff affiliated with Steno Diabetes Center will have access to the information in the patients’ journal. Other parties in the ADAPT consortium will not have access to any personally identifiable data. Review and manipulation of third party data will only take place by agreement between the subinvestigator and the primary investigator. Data will be stored for the period notified to the Danish Data Protection Agency. All biological material is destroyed at the end of the project when all analyses related to the specific research project have been completed.
